# Comparing *Mycobacterium tuberculosis* genomes using genome topology networks

**DOI:** 10.1186/s12864-015-1259-0

**Published:** 2015-02-14

**Authors:** Jianping Jiang, Jianlei Gu, Liang Zhang, Chenyi Zhang, Xiao Deng, Tonghai Dou, Guoping Zhao, Yan Zhou

**Affiliations:** State Key Laboratory of Genetic Engineering, School of Life Sciences, Fudan University, Shanghai, 200433 People’s Republic of China; Shanghai-MOST Key Laboratory of Health and Disease Genomics, Chinese National Human Genome Center at Shanghai, Shanghai, 201203 People’s Republic of China; School of Information Technology and Electrical Engineering, The University of Queensland, St Lucia, QLD 4072 Australia; Institutes of Biology and Medical Sciences, Soochow University, Suzhou, 215123 People’s Republic of China

## Abstract

**Background:**

Over the last decade, emerging research methods, such as comparative genomic analysis and phylogenetic study, have yielded new insights into genotypes and phenotypes of closely related bacterial strains. Several findings have revealed that genomic structural variations (SVs), including gene gain/loss, gene duplication and genome rearrangement, can lead to different phenotypes among strains, and an investigation of genes affected by SVs may extend our knowledge of the relationships between SVs and phenotypes in microbes, especially in pathogenic bacteria.

**Results:**

In this work, we introduce a ‘Genome Topology Network’ (GTN) method based on gene homology and gene locations to analyze genomic SVs and perform phylogenetic analysis. Furthermore, the concept of ‘unfixed ortholog’ has been proposed, whose members are affected by SVs in genome topology among close species. To improve the precision of 'unfixed ortholog' recognition, a strategy to detect annotation differences and complete gene annotation was applied. To assess the GTN method, a set of thirteen complete *M. tuberculosis* genomes was analyzed as a case study. GTNs with two different gene homology-assigning methods were built, the Clusters of Orthologous Groups (COG) method and the orthoMCL clustering method, and two phylogenetic trees were constructed accordingly, which may provide additional insights into whole genome-based phylogenetic analysis. We obtained 24 unfixable COG groups, of which most members were related to immunogenicity and drug resistance, such as PPE-repeat proteins (COG5651) and transcriptional regulator TetR gene family members (COG1309).

**Conclusions:**

The GTN method has been implemented in PERL and released on our website. The tool can be downloaded from http://homepage.fudan.edu.cn/zhouyan/gtn/, and allows re-annotating the ‘lost’ genes among closely related genomes, analyzing genes affected by SVs, and performing phylogenetic analysis. With this tool, many immunogenic-related and drug resistance-related genes were found to be affected by SVs in *M. tuberculosis* genomes. We believe that the GTN method will be suitable for the exploration of genomic SVs in connection with biological features of bacterial strains, and that GTN-based phylogenetic analysis will provide additional insights into whole genome-based phylogenetic analysis.

**Electronic supplementary material:**

The online version of this article (doi:10.1186/s12864-015-1259-0) contains supplementary material, which is available to authorized users.

## Background

Whole genome sequences can be conveniently and rapidly sequenced by next-generation sequencing (NGS) platforms, especially genomes of microorganisms, whose sizes usually range from two to six mega base pairs. One of the largest biological information centers, the National Center for Biotechnology Information (NCBI), has collected more than two thousand bacterial genomes in its Microbial Genomes Resources, and the number of microbial whole genomes submitted to NCBI is growing exponentially. These precious whole genome data may provide us with an opportunity to study genomes at a comprehensive level.

As complete genome sequences become available, genome organization studies become attractive due to their applications to gene function predictions and phylogenetic relationship studies [1]. Gene order and gene content determined by ortholog identification have been well investigated in genome organization studies. A number of previous studies have indicated that conserved gene orders may correlate with functional protein interactions [2]. As phylogenetic signals, gene order and gene content variation also play an important role in speciation and gene functional divergence. Whole genome feature-based phylogenetic methods are urgently needed as more complete genome sequences become available. In contrast to phylogenetic studies based on a single or a few genes, studies over entire genomes involve more evolutionary variations and may reveal more confident phylogenetic relationships. Some genome feature-based methods have emerged, such as using gene content, gene order and the distribution of oligonucleotides (‘DNA strings’), in phylogenetic analyses. Interestingly, a DNA string approach called the CVTree method [3] claims to be alignment-free and may have great potential in Metagenomics studies. Additionally, methods based on whole genome single nucleotide polymorphisms (SNPs) are increasingly being used in phylogenetic analysis [4].

Inspired by gene content-based and gene order-based studies in genomics, as well as network-based studies in informatics, we built a genome network called the Genome Topology Network (GTN) to study genomic structural variations (SVs) among closely related genomes. The GTN is constructed with information on gene homology relationships and gene locations, and it is less prone to homoplasy by convergence or reversal in phylogenetic studies. We also propose the concept of ‘unfixed ortholog’, whose members are affected by SVs in genome topology among close species. However, the gene annotations acquired from public databases may be inconsistent due to annotating different tools and/or parameters, making comparative studies difficult and inaccurate. To make annotations from public databases comparable and improve the 'unfixed ortholog' recognition precision, a strategy was applied to detect annotation differences between closely related genomes and to annotate remaining genes. In addition, an algorithm is provided to summarize evolutionary events between close branches in phylogenetic trees, which may provide new insights into genomic structure studies. For example, this algorithm may be used to identify strains with faster duplication or deletion rates. The GTN method has been implemented in PERL and released on our website. The current tool is tentative and relies on third-party tree-generating software. It is currently restricted to the analysis of complete genomes and will be improved to be applicable for draft genomes and support bootstrap tests in the future. To demonstrate the use of the GTN method, thirteen complete genomes of *Mycobacterium tuberculosis* were studied, observing useful and interesting results.

## Methods

### *M. tuberculosis* genome sequences, annotation refinement and orthology assignment

The genomes of thirteen *M. tuberculosis* strains were downloaded from NCBI (Table 1), and the plasmid sequences were eliminated. The annotation refinement was carried out through coding sequence (CDS) mapping by BLAST (v2.2.26) [5]; thus, the CDSs collected from the thirteen strains were used to map each genome. A new annotated gene was retrieved from external CDS that mapped 100% to an intergenic region or to a region with a less-than-80% overlap with known genes, such that pseudogenes were eliminated. Clusters of Orthologous Groups (COGs) (latest version) [6] were used as references to assign orthology, and orthoMCL (v1.4) [7] was employed to generate customized orthology clusters. Protein sequences from the thirteen *M. tuberculosis* strains were aligned to the COG database via BLAST (v2.2.26) [5] (E-value less than 1e-5), and the best hits were selected as the COG annotations. The COG clusters are referred to as the finished COG categories. The orthology clusters were determined by orthoMCL [7] with default orthology assigning parameters.

**Table 1:** **General features of thirteen** ***M. tuberculosis*** **genomes**

**Strain** **(** ***M. tuberculosis*** **)**	**Gene Bank ID**	**Source**	**CDSs**	**Added genes**	**CDSs after refinement**	**COG annotated**	$\frac{\boldsymbol{COGs}}{\boldsymbol{CDSs}}$ **(%)**	**orthoMCL Annotated**	$\frac{\mathbf{Orthologs}}{\mathbf{CDSs}}$ ** (%)**
**CCDC5079**	NC_021251.1	CHN	4,156	249	4,405	2,880	65.38	4,156	94.35
**CCDC5180**	NC_017522.1	CHN	3,590	813	4,403	2,862	65.00	4,111	93.37
**CDC1551**	NC_002755.2	USA	4,189	126	4,315	2,762	64.01	4,018	93.12
**CTRI 2**	NC_017524.1	RUS	3,944	427	4,371	2,849	65.18	4,139	94.69
**F11**	NC_009565.1	ZA	3,941	442	4,383	2,862	65.30	4,141	94.48
**H37Ra**	NC_009525.1	-	4,034	372	4,406	2,866	65.05	4,168	94.60
**H37Rv**	NC_000962.3	-	4,111	372	4,483	2,867	63.95	4,158	92.75
**KZN 1435**	NC_012943.1	ZA	4,059	325	4,384	2,859	65.21	4,148	94.62
**KZN 4207**	NC_016768.1	ZA	3,996	376	4,372	2,851	65.21	4,136	94.60
**KZN 605**	NC_018078.1	ZA	4,001	349	4,350	2,835	65.17	4,122	94.76
**RGTB327**	NC_017026.1	IN	3,691	325	4,016	2,459	61.23	3,594	89.49
**RGTB423**	NC_017528.1	IN	3,622	271	3,893	2,379	61.11	3,462	88.93
**UT205**	NC_016934.1	COL	3,796	433	4,229	2,778	65.69	4,011	94.85

Source: The strain information was acquired from NCBI. CHN: China, USA: America, RUS: Russia, ZA: South Africa, IN: India, COL: Colombia. H37Ra and H37Rv were derived from the original human-lung H37 isolate in 1934 and have been used extensively in biomedical research.

### Construction of GTNs

**Figure 1:**
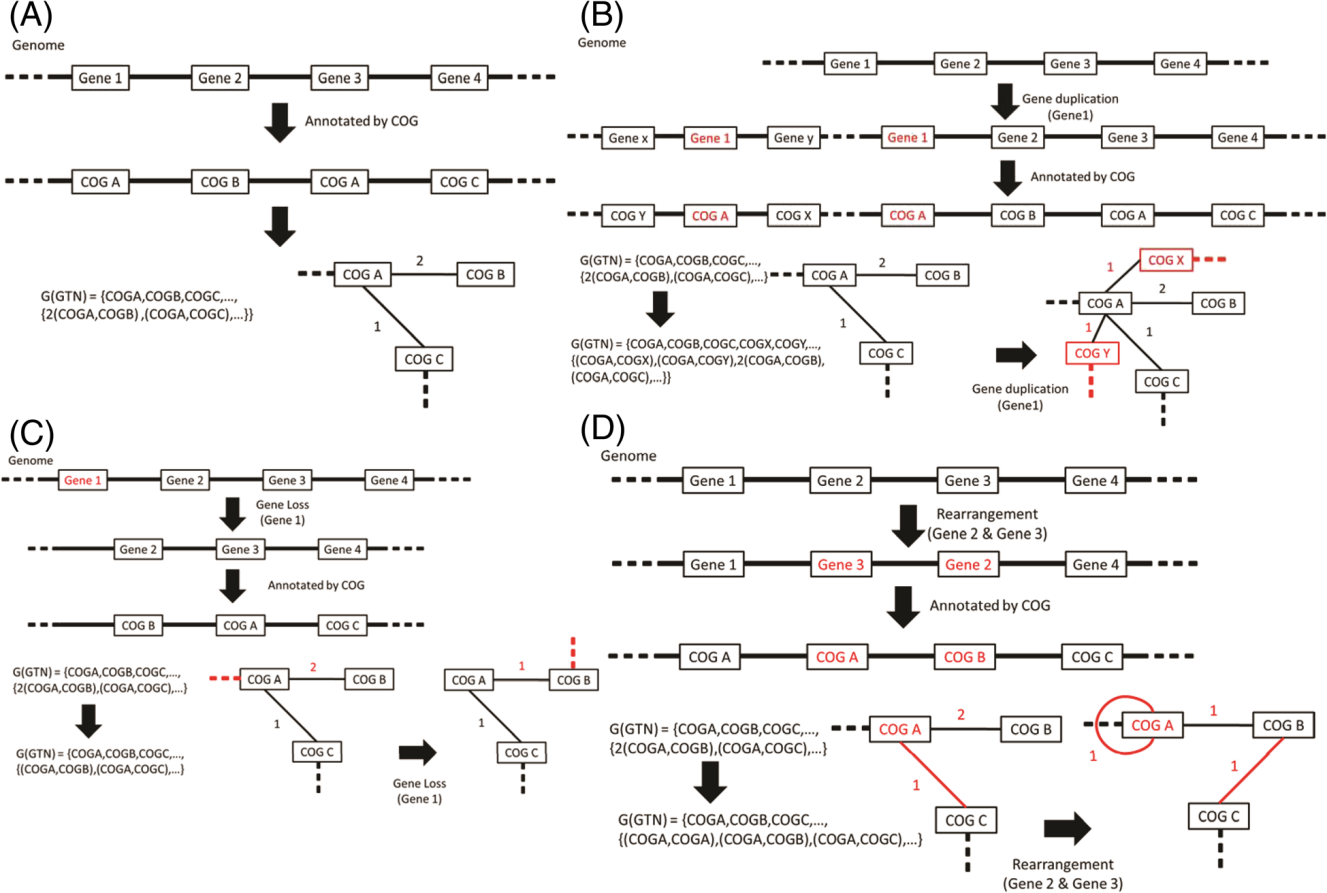
**The construction of GTNs and how GTNs are affected by evolutionary events. (A)** Constructing a GTN from a genome. Supposing that Gene1 and Gene3 are annotated as COGA, Gene2 as COGB and Gene4 as COGC, the numbers adjacent to the lines in the GTNs are degrees. **(B)** The variation in GTN structure when Gene1 is duplicated and inserted between GeneX and GeneY. GeneX and GeneY are annotated as COGX and COGY, respectively. **(C)** The variation in GTN structure when Gene1 is lost. **(D)** The variation in GTN structure when the segments of Gene2 and Gene3 are reversed in the genome.

A GTN is described as **G(GTN) = <V(GTN), E(GTN) >** (Figure 1). A **G(GTN)** contains a vertex set **V(GTN)** and an edge set **E(GTN)**. The vertex set is an orthologous set found in a genome and is described as **V(GTN) = {OrthoA, OrthoB, OrthoC, …}** in a GTN. The edge set is an undirected edge set containing edges of all vertices in a GTN. The edge set is described as **E(GTN) = {e**_**1**_**,e**_**2**_**,e**_**3**_**,..}**, where each element (e1, e2, etc.) represents a pair of adjacent genes (orthologs) in the genome. The vertexes are determined by orthologous clustering, and the edges are determined by the gene order. To illustrate, COG-based GTNs were taken. Supposing that a genome contains four genes, g1, g2, g3 and g4, rank orderly in the genome. Among them, g2 and g3 do not have hits in the COG database, and g1 and g4 are annotated as COGA and COGB, respectively. Then, the vertex set of the genome is V(G) = {COGA,COGB}, and the edge set is E(G) = {e_COGA, COGB_}. Notably, a circular bacterial genome is treated as a linear structure beginning with *dnaA* here.

The degree of an edge **e** is the frequency of the ortholog pairs in the GTN and is described as **Degree(e) = Frequency(e)**; for example, if the edge e_COGA, COGB_ appears twice in a genome, the degree of this edge is 2 and is described as Degree(e_COGA,COGB_) = 2. If a genome has **n** COG-annotated genes and is clustered into **m** COG groups, then the vertex number of this GTN is **m**, and the sum of the degrees in this GTN is **n-1**.

### Unfixed gene definition and enrichment analysis of an unfixed gene set

The unfixed ortholog is defined before the unfixed gene. An unfixed ortholog is determined by the different degree of the ortholog between two genomes (p and q). The formula of the different degree (**DD**) is:

1 $$ {\mathrm{DD}}_{p,q}\left( Ortho\ I\right) = \mathrm{ROUNDDOWN}\left({\displaystyle \sum}\left| degree{\left({e}_{Ortho\ I,\  Ortho\ i}\right)}_p- degree{\left({e}_{ortho\ I,\  Ortho\ i}\right)}_q\right|\right) $$

The ***Ortho I*** in equation (1) denotes the target ortholog, and the ***Ortho i*** represents all the orthologs that are adjacent to ***Ortho I*** in genomes p and q. The result of formula (1) reflects the different constitutions of adjacent genes of ***Ortho I*** on the two genomes. An ortholog group containing more genes affected by SVs would acquire a higher DD. If the result of equation (1) is greater than 0, the orthologous group is regarded as an unfixed ortholog, and the orthologous group’s genes are defined as unfixed genes (Additional file 1: Figure S1).

When the genome number is **N** (**N** > 2), the average DD (DD_ave_) calculated by the following formula will be used:

2 $$ {\mathrm{DD}}_{ave}\left( Ortho\ I\right) = \mathrm{A}\mathrm{V}\mathrm{E}{\left[D{D}_{p,\ q}\left( Ortho\ I\right)\right]}_{p\ne q} $$

In the unfixed gene enrichment analysis, the COG database was used to determine orthologous relationships. COGs are classified into twenty-one sub-categories in the COG database. The enrichment analysis was conducted by Fisher’s exact test for each sub-category, and the null hypothesis was that the proportion of unfixed COG groups is higher (or lower) in one sub-category than in the other sub-categories. The sub-category whose P-value is less than 0.05 will be considered to possess significantly more (or fewer) unfixed COG groups than other sub-categories.

### The calculation of the distance between GTNs and phylogenetic analysis

Distance is used to describe the difference between two GTNs, and the distance between two GTNs constructed with Genome1 and Genome2 is defined as follow:

3 $$ \mathrm{D}\left({\mathrm{G}}_1,{\mathrm{G}}_2\right)=1-\frac{1}{\mathrm{N}}{\displaystyle {\sum}_{i=1}^N}\frac{2\times \mathrm{Ci}}{\uptau_1\mathrm{i} + {\uptau}_2\mathrm{i}} $$

In the formula, **N** is the number of total non-redundant vertexes of Genome1 and Genome2. τ_1_i is the number of vertexes (orthologs) that are adjacent to vertex i (ortholog i) in Genome1, and τ_2_i is the number of vertexes that are adjacent to vertex i in Genome2. Ci is the number of common vertexes that are adjacent to vertex i (ortholog i) in the two genomes. In formula (3), a cumulative ***Jaccard*** distance that is frequently used in measuring the difference between two objects is used to weigh the difference between two GTNs.

The two distance matrices are calculated with orthologous relationships determined by COG and orthoMCL among thirteen *M. tuberculosis* genomes and the *M. bovis* BCG genome. The *M. bovis* BCG used in the phylogenetic analyses is the outgroup. In total, 57 housekeeping genes shared among thirteen *M. tuberculosis* strains and *M. bovis* BCG [8] were used in the multi-gene tree construction. All phylogenetic trees were inferred using the neighbor-joining (NJ) method with the MEGA (v5.2.2) software [9]. In the construction of the multi-gene tree, the bootstrap method was used to test the confidence of topology.

### Lost gene recognition and enrichment analysis of the lost genes

The lost genes are obtained by comparison of orthologs identified by orthoMCL between a strain and the reference strain H37Rv. If orthologs exist in H37Rv and are absent in the query strain, they are considered lost genes. The KEGG [10] pathway enrichment analyses were carried out on lost gene sets using the online tool DAVID [11]. The enrichment analysis background is the set of genes in the reference strain H37Rv.

## Results

### Annotation refinement and orthology assignment

We found that genome annotation accuracy was important for GTN construction and unfixed gene recognition. However, many genes were left unannotated in some genomes due to using different annotation tools and annotation parameters. For instance, a comparison of the annotations in two well-annotated strains, H37Ra and H37Rv, shows that the different content and order of COG4118 (antitoxin of toxin-antitoxin stability system, Phd gene family) is a false positive. Both H37Ra and H37Rv possess seven COG4118 paralogs; however, only six pairs are identical between the two genomes. After mapping H37Ra’s CDSs to the H37Rv genome, an unannotated COG4118 orthologous gene termed MRA_2898 was observed in H37Ra at a similar location in H37Rv and vice-versa for the H37Rv gene Rv2830c. The gene overlaps should be responsible for the annotation inconsistency (Additional file 1: Figure S2). After annotation refinement, H37Ra and H37Rv were shown to possess eight COG4118 paralogs.

**Figure 2:**
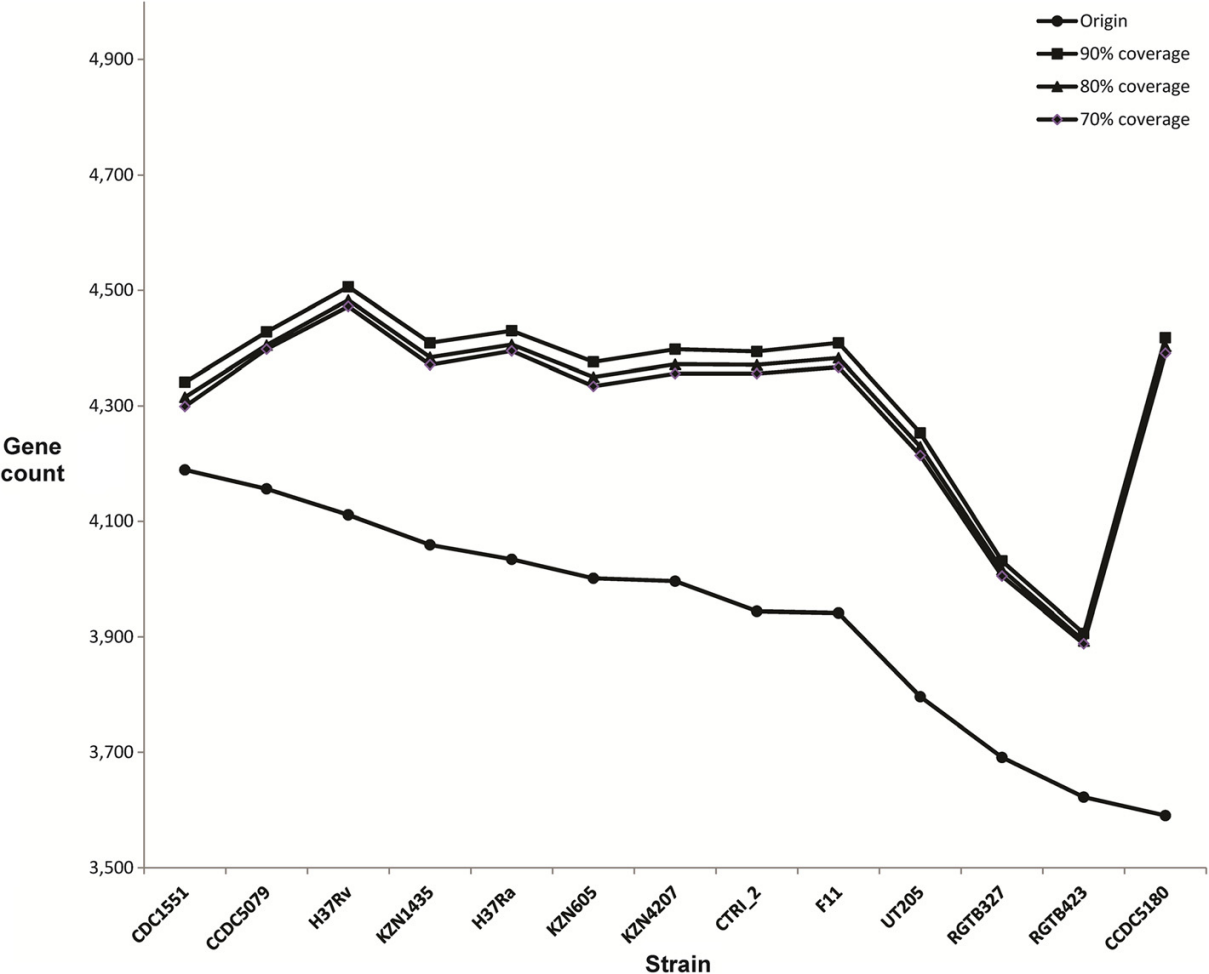
**Gene counts of thirteen** ***M. tuberculosis*** **strains after annotation refinement with different overlap thresholds.** The horizontal axis shows each of the thirteen *M. tuberculosis* strain names, and the vertical axis depicts the total gene count in each strain. After annotation refining with different overlap thresholds (70%, 80% and 90%), the gene count of each strain increased. However, when the threshold was set to 70% or 90%, there were few differences in the results.

The gene overlap among thirteen strains reveals that gene overlap commonly exists in original gene annotations (Additional file 1: Figure S6). The largest overlap ratio in the original annotations, the overlap between MTCTRI2_3084 and MTCTRI2_3085 in CTRI-2, was near 100%. The threshold of the overlap ratio in CCDC5180 was less than 20%, which was much lower than that of other strains. CDC1551 possesses the most overlapped genes, nearly twofold more than the other strains. Due to the importance of gene overlap in annotations, different overlap thresholds were used in the annotation refinement. However, when the threshold was set to 70% or 90%, there were few differences in the results. Thus, the overlap threshold between the additionally annotated genes and the known genes was set to less than 80% in the final annotation refinement. After annotation refinement, the overlapped genes increased in every strain, and both the number of overlapped genes and the overlap ratio share similar distributions within the thirteen strains (Additional file 1: Figure S7). In total, 126 to 813 new annotated genes were added to the thirteen strains (Table 1, Figure 2). The total gene number in each strain was approximately 4,400, with the highest being 4,483 (H37Rv) and the lowest ranging from 3,900 to 4,000 (RGTB423, RGTB327). Most of the *M. tuberculosis* genes (61.11% to 65.69%) are annotated by the COG reference sequences. The majority of the genes (88.93% to 94.85%) are clustered and assigned orthologous groups by orthoMCL.

### Unfixed COG groups

The refined genes are clustered into 1,414 COG groups, and more than three-fourths (1,113 COGs) have DDs (defined in methods) greater than 0, indicating the prevalence of genomic SVs (Additional file 1: Figure S3). The enrichment analysis between unfixed COG groups and fixed COG groups (Additional file 1: Table S1) in each COG sub-category shows that the sub-category of ‘secondary metabolites biosynthesis, transport and catabolism’ contains significantly more unfixed COGs (P-value < 0.05). The COG groups with the most DDs are listed in Table 2. Interestingly, the PPE-repeat proteins (COG5651) group, which is described as encoding virulence factors and antigens, has the highest DD (31). The PPE-repeat proteins group containing more than sixty paralogs is highly polymorphic and is thought to be the main source of complex antigenic variation in *M. tuberculosis* [12]. As expected, four types of transposase are found with high DDs. Additionally, the transcriptional regulator TetR family (COG1309) and three groups, *fadD* COG0318, *fadE* COG1960 and *echA* COG1024, which are involved in lipid β-oxidation, possess high DDs.

**Table 2:** **COG groups with the highest DDs**

**COG ID**	**Annotation**	**DD/Str.**	**Paralog/Str.**	**DD/Paralog**
**COG2963**	Transposase and inactivated derivatives	24	14	1.71
**COG2801**	Transposase and inactivated derivatives	28	18	1.56
**COG3547**	Transposase and inactivated derivatives	6	6	1.00
**COG3328**	Transposase and inactivated derivatives	5	9	0.56
**COG5651**	PPE-repeat proteins	31	62	0.50
**COG2114**	Adenylate cyclase, family 3 (some proteins contain HAMP domain)	6	12	0.50
**COG0515**	Serine/threonine protein kinase	5	11	0.45
**COG1680**	Beta-lactamase class C and other penicillin-binding proteins	5	11	0.45
**COG0277**	FAD/FMN-containing dehydrogenases	6	14	0.43
**COG2409**	Predicted drug exporters of the RND superfamily	6	14	0.43
**COG3321**	Polyketide synthase modules and related proteins	8	19	0.42
**COG1024**	Enoyl-CoA hydratase/carnitine racemase	9	23	0.39
**COG0657**	Esterase/lipase	5	13	0.38
**COG0596**	Predicted hydrolases or acyltransferases (alpha/beta hydrolase superfamily)	12	34	0.35
**COG3315**	O-Methyltransferase involved in polyketide biosynthesis	6	17	0.35
**COG1309**	Transcriptional regulator	17	49	0.35
**COG0318**	Acyl-CoA synthetases (AMP-forming)/AMP-acid ligases II	11	32	0.34
**COG1960**	Acyl-CoA dehydrogenases	12	35	0.34
**COG2141**	Coenzyme F420-dependent N5,N10-methylene tetrahydromethanopterin reductase and related flavin-dependent oxidoreductases	6	19	0.32
**COG2124**	Cytochrome P450	6	20	0.30
**COG0477**	Permeases of the major facilitator superfamily	8	27	0.30
**COG1848**	Predicted nucleic acid-binding protein, contains PIN domain	5	19	0.26
**COG0500**	SAM-dependent methyltransferases	9	37	0.24
**COG1028**	Dehydrogenases with different specificities (related to short-chain alcohol dehydrogenases)	9	41	0.22

DD/Str.: The averages of different degrees. The COG group’s average DDs higher than five are shown; Paralog/Str.: The average paralog number (rounded) of thirteen *M. tuberculosis* strains.

### Phylogenetic trees

**Figure 3:**
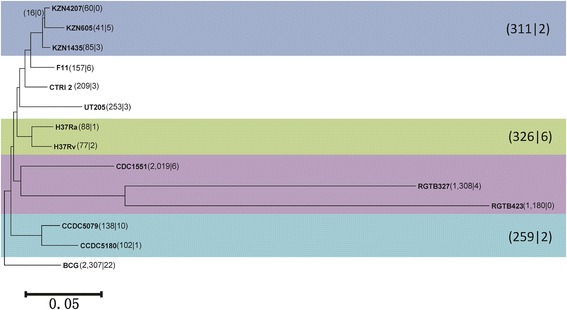
**A rooted phylogenetic tree constructed with orthologs determined by orthoMCL of thirteen** ***M. tuberculosis*** **stains and the outgroup** ***M. bovis*** **BCG.** The strain information is shown in Table 1. The first number in parentheses is the number of unique ortholog pairs in the strain when compared to its sister group. The second number corresponds to the differences of existing ortholog pairs in a strain when compared to its sister group. For instance, there are 60 unique ortholog pairs in KZN4207 compared with KZN605. There are 41 unique ortholog pairs in KZN605. For the ortholog pairs in both strains (groups), five more existences were detected in KZN4207 than in KZN605. The sub-groups are marked with different colors. The blue background denotes the KZN group, green denotes the H37 group, purple denotes the RGTB group and light blue denotes the CCDC group. The RGTB group was excluded from the group due to its abnormal gene counts. The number to the right of each group is the number of different ortholog pairs (unique and different) between the group and the other two groups.

The two phylogenetic trees (Figure 3 and Additional file 1: Figure S4) constructed with COG GTNs and orthoMCL GTNs show great similarity in topology. The phylogenetic analysis of orthoMCL GTNs results in a rooted phylogenetic tree grouping fourteen strains into 3 lineages: (i) BCG, (ii) the Beijing family and (iii) the non-Beijing family. The topology of the COG GTN tree is nearly identical to that of the orthoMCL GTN tree (Additional file 1: Figure S4). A comparison of the two GTN-based trees with the multi-gene tree constructed with 57 housekeeping genes (Additional file 2: Figure S5) shows that the topologies are similar at the confident branches (bootstrap value > 90%). The SV study shows that few ortholog pair variations occur within the KZN group (blue), H37 group (green) or CCDC group (light blue) (Figure 3). There are 311, 326 and 259 unique ortholog pairs in the KZN group, the H37 group and the CCDC group, respectively, when each is compared to the other two groups. The ortholog pair variations mostly occur in the RGTB group (purple) in *M. tuberculosis*, and CDC1551 possesses 2,019 unique ortholog pairs compared to its sister groups, RGTB327 and RGTB423.

### Enrichment analysis of lost gene sets in three strains

The GTN tree-based ortholog pair variation analysis reveals that three strains, RGTB327, RGTB423 and CDC1551, hold more pair variations than other strains. There were 685, 599 and 166 genes lost in RGTB423, RGTB327 and CDC1551, respectively, compared to H37Rv (Table 3). The lost genes from RGTB327 show enrichment in eight pathways involved in metabolism, biosynthesis and degradation. Among the enriched pathways, the ABC transporters may play important roles in drug resistance [13]. The lost genes from RGTB423 were enriched in twenty pathways; among them, fatty acid metabolism is a key pathway during infection [14], and the two-component system pathway is important in the immune response [15]. The lost genes from CDC1551 show enrichment in three pathways, and the lipopolysaccharide biosynthesis pathway is important to endotoxin bioactivity [16]. RGTB423 and RGTB327 share five enriched pathways, while CDC1551 shares no enriched pathways with RGTB423 and RGTB327.

**Table 3:** **Pathway (KEGG) enrichment analysis of lost genes from three strains**

**Strain** **(** ***M. tuberculosis*** **)**	**Total Lost** **(vs. H37Rv)**	**Hits**	**Pathway**	**P-value**
**RGTB327**	**599**	17	**mtu00230:Purine metabolism**	0.00
		12	mtu00240:Pyrimidine metabolism	0.01
		10	mtu00361:gamma-Hexachlorocyclohexane degradation	0.01
		13	**mtu00624:1- and 2-Methylnaphthalene degradation**	0.00
		16	**mtu00632:Benzoate degradation via CoA ligation**	0.01
		16	**mtu00903:Limonene and pinene degradation**	0.01
		5	**mtu01053:Biosynthesis of siderophore group nonribosomal peptides**	0.01
		16	mtu02010:ABC transporters	0.00
**RGTB423**	**685**	15	mtu00071:Fatty acid metabolism	0.00
		16	**mtu00230:Purine metabolism**	0.01
		10	mtu00250:Alanine, aspartate and glutamate metabolism	0.00
		8	mtu00260:Glycine, serine and threonine metabolism	0.04
		18	mtu00280:Valine, leucine and isoleucine degradation	0.00
		19	mtu00281:Geraniol degradation	0.00
		13	mtu00330:Arginine and proline metabolism	0.00
		7	mtu00360:Phenylalanine metabolism	0.03
		15	mtu00380:Tryptophan metabolism	0.00
		10	mtu00410:beta-Alanine metabolism	0.04
		6	mtu00480:Glutathione metabolism	0.03
		13	**mtu00624:1- and 2-Methylnaphthalene degradation**	0.01
		16	**mtu00632:Benzoate degradation via CoA ligation**	0.03
		19	mtu00640:Propanoate metabolism	0.00
		16	**mtu00903:Limonene and pinene degradation**	0.03
		8	mtu00910:Nitrogen metabolism	0.01
		9	mtu00930:Caprolactam degradation	0.04
		6	**mtu01053:Biosynthesis of siderophore group nonribosomal peptides**	0.00
		16	mtu02020:Two-component system	0.00
		7	mtu03030:DNA replication	0.01
**CDC1551**	**166**	4	mtu00310:Lysine degradation	0.01
		2	mtu00540:Lipopolysaccharide biosynthesis	0.03
		4	mtu00650:Butanoate metabolism	0.02

Pathway: KEGG pathways are enriched in lost genes. Shared pathways between RGTB327 and RGTB423 are shown in bold.

## Discussion

In this study, a network-based method was raised to analyze the structural dynamics of genomes. To illustrate the application of this method, we analyzed thirteen complete *M. tuberculosis* genomes and discovered much useful information. The tool exhibits the following functions: gene annotation refinement, orthology assignment with COG, unfixed gene analysis and phylogenetic analysis. Furthermore, orthologous relationships can be determined by any orthology assigning method.

The consistency of gene annotation is crucial in GTN construction, as gene content and gene order may affect the framework of GTN. During orthology assignment, we find that a number of genes have not been properly annotated in some strains. Comparing annotations from H37Rv and H37Ra shows that annotation refinement should be applied to thirteen *M. tuberculosis* strain annotations. The goal of the annotation refinement was to minimize the impact of annotation inconsistency and improve the precision of 'unfixed ortholog' recognition. The phenomenon of many genes left unannotated in CCDC5180 demonstrates that longer overlaps might be taken into account during the annotation of *M. tuberculosis* genomes. Despite performing annotation refinement, the gene numbers of RGTB327 and RGTB423 still seem abnormal when compared to those of other strains. Furthermore, the records of RGTB327 and RGTB423 in NCBI were marked as ‘removed’ recently, indicating that the authors and/or the staff may realize the imprecision of the data. The minor differences in the results after annotation refinement under varied overlap thresholds show that the refining method is robust in terms of overlaps between additional and known annotations.

Whole genome alignment-based methods can provide a visual view of SVs among multiple genomes. However, the excessive attention paid to the alignment may hamper the exploration of biological issues via sequence-oriented methods. Our network-based method is function-oriented and easy to use. It has shown potential for exposing drug resistance and pathogenesis-related genes affected by SVs. In the study of thirteen *M. tuberculosis* strains, enrichment analysis of unfixed COG groups show that the ‘translation, ribosomal structure and biogenesis’ category contains fewer unfixed COGs, which may indicate the conservation of its members. More COG groups in ‘secondary metabolites biosynthesis, transport and catabolism’ possess high DDs, which may indicate that these members are active in the thirteen *M. tuberculosis* strains.

The unfixed gene analysis of the thirteen strains resulted in 24 top COG groups with high DDs. The transposases (COG2963, COG2801, COG3547 and COG3328) and the PPE-repeat proteins (COG5651) shown to be highly polymorphic in previous studies have high DDs in our study. Some COG groups with high DDs are related to energy and lipid metabolism, such as *fadD* (COG0318), *fadE* (COG1960) and *echA* (COG1024). In *M. tuberculosis*, fatty acids degradation is the main energy source during infection, and lipid metabolism plays an important role in invading host cells [14]. *fadE* is up-regulated when exposed to INH [17], and the expression of *echA* increases when the bacteria are exposed to macrophages [18]. It is interesting that the *fadD* gene becomes up-regulated when treated with SDS [17]. Some COG groups are related to secondary metabolism, such as ‘polyketide synthase modules and related proteins’ (COG3321) and ‘O-methyltransferase involved in polyketide biosynthesis’ (COG3315). The *pks* and *pps* genes are grouped in COG3321, and the deletion of *pks12* has shown a drug resistance incensement in *Mycobacterium avium* [19]. Zheng, et al. also showed that mutations in *pks12* may be responsible for the loss of virulence in H37Ra [20]. Furthermore, we found that the gene *pks3* from H37Ra encoding 2,155 amino acids aligned to two genes, *pks3* and *pks4*, encoding 488 and 1,582 amino acids, respectively, from H37Rv. H37Rv contains a stop codon at base pair 1,315,189, between *pks3* and *pks4*. Some COG groups involved in gene regulation have high DDs, such as members of the TetR family (COG1309). The TetR family proteins are involved in the transcriptional control of multidrug efflux pumps, the biosynthesis of antibiotics, responses to osmotic stress and toxic chemicals, differentiation and pathogenicity [21]. The SV of transcriptional regulators may result in changes in the expression of target genes. For example, the expression of *acrEF* in *Escherichia coli* increases after a gene is inserted upstream [22]. *M. tuberculosis* contains three members of the TetR family. One of the members (Rv3066) controls the expression of *Mmr*, which functions as a multidrug efflux pump [23]. Some COG groups relating to signal transduction possess high DDs, such as ‘adenylate cyclase’ (COG2114). Interestingly, the relative DD of adenylate cyclase is equal to that of PPE repeat-proteins and is higher than those of most other groups. Adenylate cyclase has been shown to facilitate the delivery of bacterial-derived cyclic AMP (cAMP) into the macrophage cytoplasm and lead to cAMP intoxication in macrophages [24]. There are many drug-related COG groups possessing high DDs, such as ‘cytochrome P450’ (COG2124), ‘coenzyme F420-dependent N5, N10-methylene tetrahydromethanopterin reductase and related flavin-dependent oxidoreductases’ (COG2141) and ‘predicted drug exporters of the RND superfamily’ (COG2409). Previous comparative genomic analysis of H37Ra and H37Rv has shown that a member of COG2409 named *MmpL14* is unique to H37Rv, and *MmpL14* is predicted to be a transmembrane transport protein. Furthermore, IS elements have been detected flanking *MmpL14* [20]. In *M. tuberculosis,* cytochrome P450 enzymes are potential candidates for drug targeting [25], and the possession of high DD may be due to the wide use of anti-tuberculosis drugs.

The increasing number of publicly accessible genome sequences makes it possible to reconstruct phylogenetic trees based on whole genome features. Previous studies have suggested little diversity in *M. tuberculosis* genomes [26]; however, evolutionary events were detected in our study, supporting the use of a GTN-based tree to elucidate whole genome topology changes and implying potential application on a general species level. The method will be applied to other bacteria and eukaryotes in the future. In the phylogenetic analysis, ortholog pair variations were used as phylogenetic signals to reveal evolutionary histories. Ortholog pair variations may not directly reflect evolutionary events, but they are correlated.

The COG-GTN and orthoMCL-GTN trees, which include more than 61% and 88% of *M. tuberculosis* genes, respectively, are nearly similar, with the exception of UT205, suggesting the robustness of the GTN approach for gene numbers and its potential usage in other scenarios. However, the current method is tentative and relies on third-party tree-generating software. We will improve the method to support bootstrap tests in the future. Some parts of the multi-gene based tree are low confidence, suggesting more sequences are needed to provide sufficient phylogenetic signals. Previous studies have shown that whole genome SNP-based approaches are a reliable method by which to infer phylogenies in *M. tuberculosis*, and six lineages have been classified in the *M. tuberculosis* complex [27,28]. In this study, two strains of the Beijing family, CCDC5079 and CCDC5180, belonging to lineage 2, eleven strains of non-Beijing family, belonging to lineage 4, and the BCG were used. Two strains, H37Rv and BCG, are shared with the previously published SNP-based tree. The GTN tree topology agrees with the hypothesis that the geographic origin is a remarkable factor affecting *M. tuberculosis* strain genome structures [29]. For example, the F11 branch, isolated from Western Cape (South Africa), is closely related to the cluster of three strains isolated from KwaZulu-Natal (South Africa). The tree also shows that H37Ra is a sister group of H37Rv, which agrees with the evolutionary histories of H37Rv and H37Ra. According to variation events, the strains CDC1551, RGTB327 and RGTB423, belonging to the non-Beijing family, possess notable differences in gene content, suggesting that they have undergone (or are undergoing) a period with a fast deletion rate, if the data of the two RGTBs were reliable.

## Conclusions

We developed a network-based method, GTN, to investigate structural dynamics of bacterial genomes. The method was implemented in PERL and released on our website. The tool makes identification of genes affected by SVs and phylogenetic analysis easy. To assess the GTN method, thirteen complete genomes of *M. tuberculosis* were studied, and useful and interesting results were obtained. Some immunogenic-related and drug resistance-related genes were showed to be affected by SVs in *M. tuberculosis*; for instance the PPE repeat genes and the *pks* genes are affected in this way. The phylogenetic analyses of *M. tuberculosis* and *M. bovis* BCG were based on the GTN result of two rooted trees, which were compatible with the published SNP phylogenetic tree. Our network-based method is function-oriented and easy to use. It has shown potential for exposing drug resistance and pathogenesis-related genes in *M. tuberculosis*. The GTN method is expected to be further used in other species.

## Additional files

Additional file 1: Table S1. Enrichment analysis of unfixed COG groups from 13 *M. tuberculosis* strains. **Figure S1.** Examples of different degree (DD) calculations in two reduced GTNs. **Figure S2.** Distribution of genes from COG4118 (black bar) in H37Ra and H37Rv. **Figure S3.** The counts of unfixed COG groups at different levels of DD in thirteen *M. tuberculosis*. **Figure S4.** A rooted phylogenetic tree constructed with orthologs assigned by the COGs of 13 *M. tuberculosis* stains and the outgroup *M. bovis* BCG. **Figure S6.** The distribution of original annotation overlaps in thirteen *M. tuberculosis* strains. **Figure S7.** The distribution of overlaps after annotation refinement in thirteen *M. tuberculosis.*
Additional file 2: Figure S5. Phylogenetic analysis of fifty-seven housekeeping genes shared within 13 *M. tuberculosis* stains and *M. bovis* BCG.
