# Supplementary material for: Comparing Mycobacterium tuberculosis genomes using genome topology networks
Source: BMC Genomics. 2015 Feb 14;16(1):85. doi: 10.1186/s12864-015-1259-0 (PMC4342819; doi:10.1186/s12864-015-1259-0)

# Tables

## Table S1- Enrichment analysis of unfixed COG groups from 13 *M. tuberculosis* strains

| Classification | COG  No. | Unfixed COG No. | $\frac{\boldsymbol{COG No.}}{\boldsymbol{Mobile No.}}\boldsymbol{(\%)}$ | | P-Value |
| --- | --- | --- | --- | --- | --- |
| CELLULAR PROCESSES AND SIGNALING | **212** | **173** | **81.60** |  | |
| Cell cycle control, cell division, chromosome partitioning | 18 | 17 | 94.44 | 0.15 | |
| Cell motility | 3 | 3 | 100.00 | 1.00 | |
| Cell wall/membrane/envelope biogenesis | 60 | 47 | 78.33 | 0.87 | |
| Defense mechanisms | 15 | 13 | 86.67 | 0.75 | |
| Intracellular trafficking, secretion, and vesicular transport | 23 | 19 | 82.61 | 0.80 | |
| Posttranslational modification, protein turnover, chaperones | 58 | 44 | 75.86 | 0.51 | |
| Signal transduction mechanisms | 35 | 30 | 85.71 | 0.41 | |
| INFORMATION STORAGE AND PROCESSING | **296** | **219** | **73.99** |  | |
| Replication, recombination and repair | 99 | 82 | 82.83 | 0.44 | |
| RNA processing and modification | 1 | 1 | 100.00 | 1.00 | |
| Transcription | 71 | 56 | 78.87 | 1.00 | |
| Translation, ribosomal structure and biogenesis | 125 | 80 | 64.00 | <0.01* | |
| METABOLISM | **640** | **521** | **81.15** |  | |
| Amino acid transport and metabolism | 134 | 111 | 82.84 | 0.32 | |
| Carbohydrate transport and metabolism | 75 | 61 | 81.33 | 0.77 | |
| Coenzyme transport and metabolism | 102 | 79 | 75.96 | 0.38 | |
| Energy production and conversion | 109 | 84 | 77.06 | 0.54 | |
| Inorganic ion transport and metabolism | 71 | 57 | 80.28 | 0.88 | |
| Lipid transport and metabolism | 53 | 46 | 86.79 | 0.23 | |
| Nucleotide transport and metabolism | 57 | 46 | 80.70 | 0.87 | |
| Secondary metabolites biosynthesis, transport and catabolism | 39 | 37 | 94.87 | 0.01* | |
| POORLY CHARACTERIZED | **359** | **280** | **78.43** |  | |
| Function unknown | 178 | 126 | 71.19 | 0.01* | |
| General function prediction only | 181 | 154 | 85.56 | 0.02* | |
| Sum | 1,507 | 1,193 |  |  | |

* Significant difference.

# Figures

## Figure S1- Examples of different degree (DD) calculations in two reduced GTNs. We suppose COGA, COGB, COGC and COGD are the COG groups in GTNP and GTNQ. The DDs of COGA in situation I and situation II are larger than 0; thus, COGA in situation I and situation II is considered an unfixed COG group. The DD of COGA in situation II is greater than that of situation I. COGA in situation II is considered more unfixable than that of situation I. The DD of COGA in situation III is equal to zero. COGA in situation III is not considered an unfixed COG group.


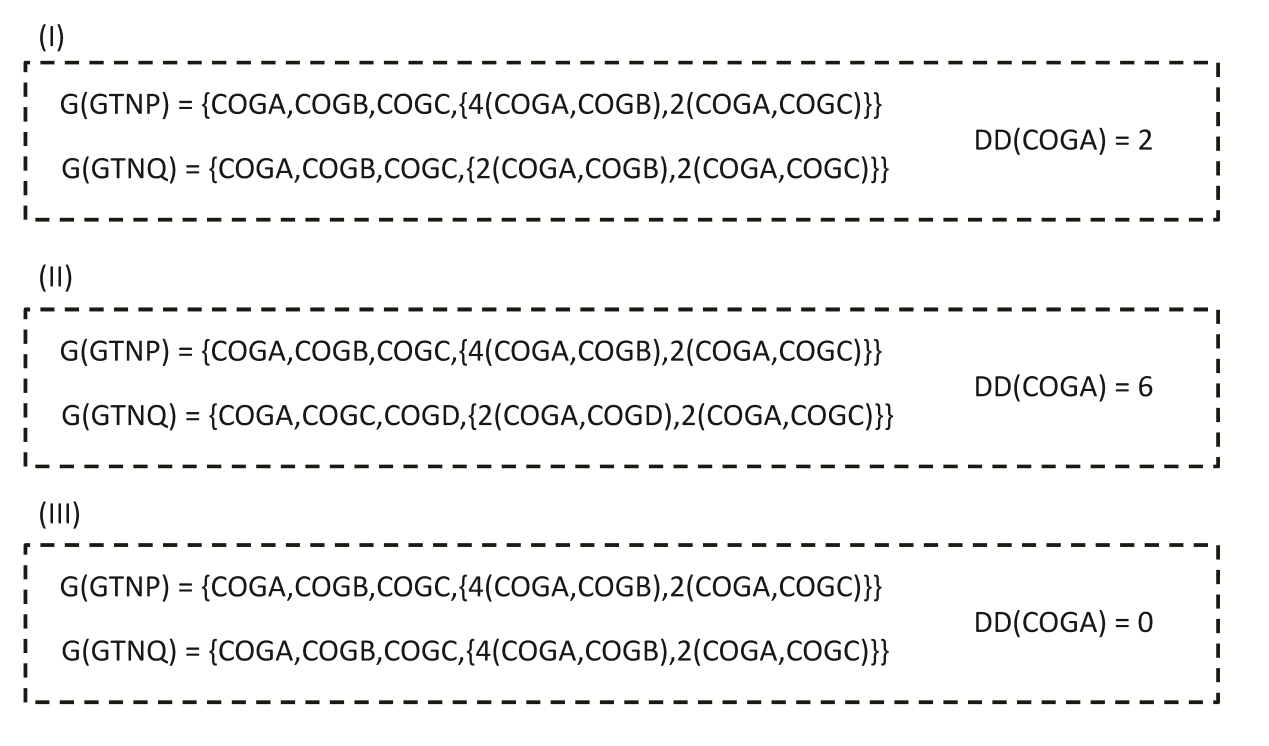


## Figure S2 - Distribution of genes from COG4118 (black bar) in H37Ra and H37Rv. (A) Map of gene content and gene order of genes annotated with COG4118 in H37Ra and H37Rv. H37Ra and H37Rv are unique genes found in each other’s genomes. (B) There is a region that is identical to an H37Rv unique gene in H37Ra, and this region has a 47-base pair (bp) overlap with an upstream gene. (C) The H37Ra unique gene is found in H37Rv, and the region that is identical to the H37Ra unique gene has an 8 bp overlap with its downstream gene.


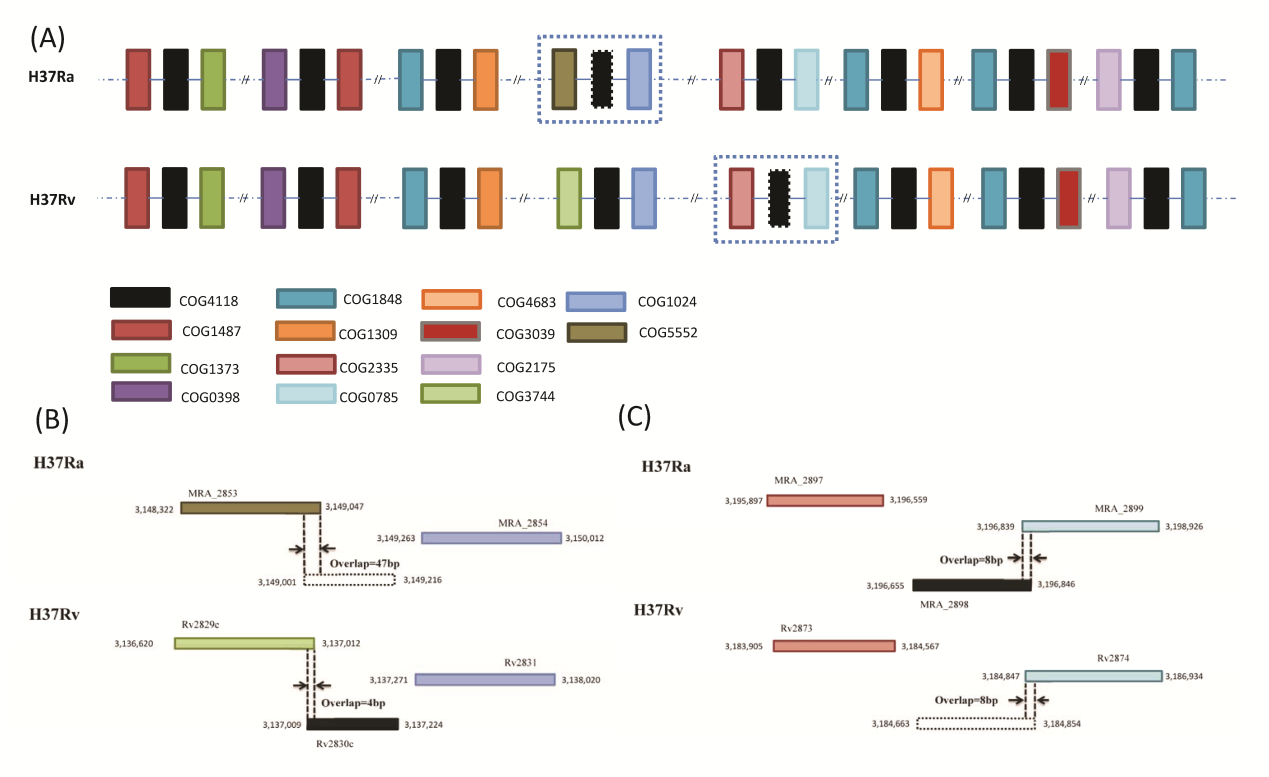


## Figure S3 - The counts of unfixed COG groups at different levels of DD in thirteen *M. tuberculosis*. The horizontal axis represents the DD, and the vertical axis represents the total COG count in each group. The majority of the DDs that the unfixed genes possessed are equal to one. The highest DD is 31.


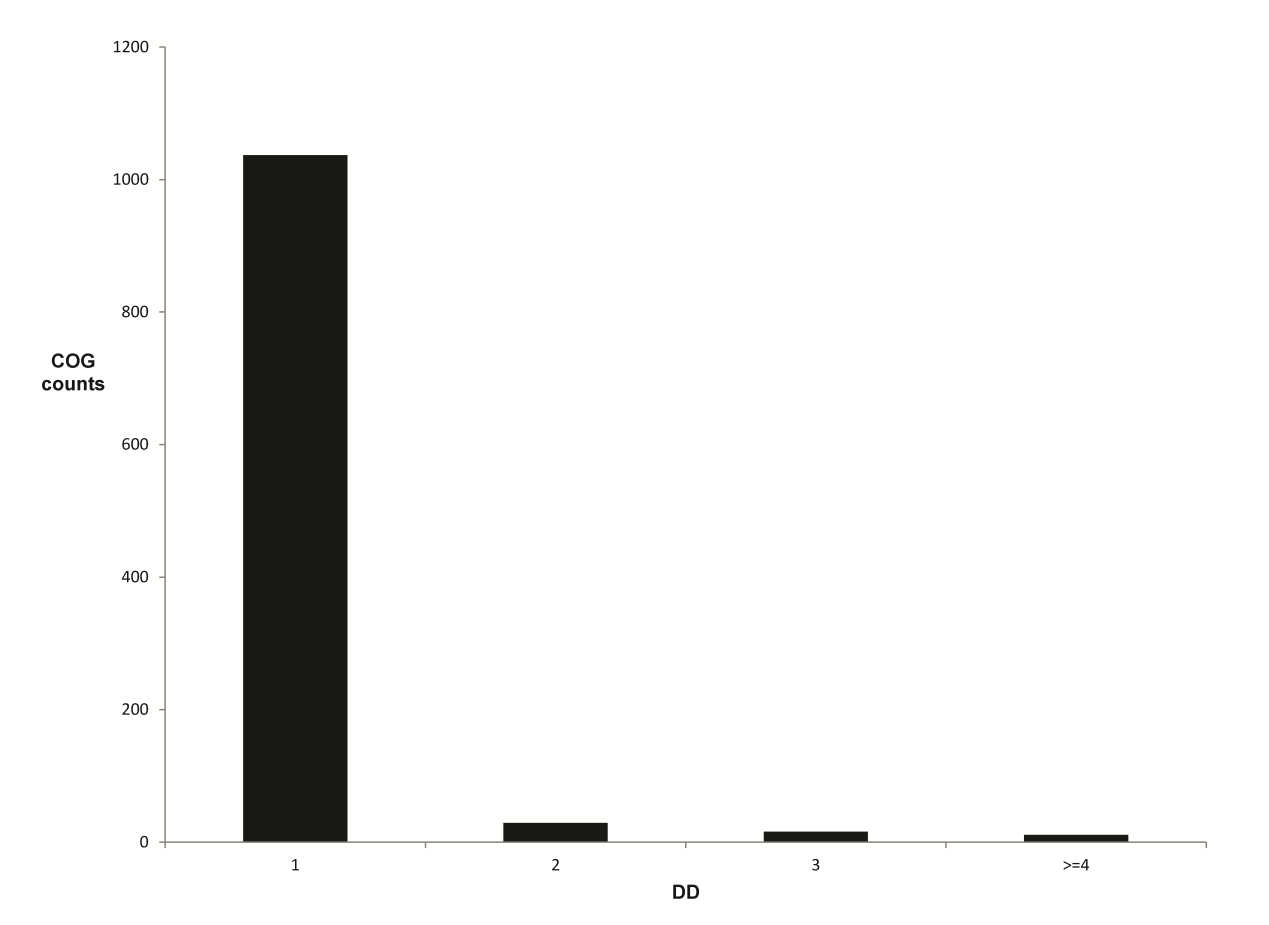


## Figure S4 - A rooted phylogenetic tree was constructed with orthologs assigned by the COGs of 13 *M. tuberculosis* stains and the outgroup *M. bovis* BCG. The first number in parentheses is the number of unique COG pairs in the strain when compared to its sister group. The second number corresponds to the differences among existing COG pairs in a strain compared to its sister group.


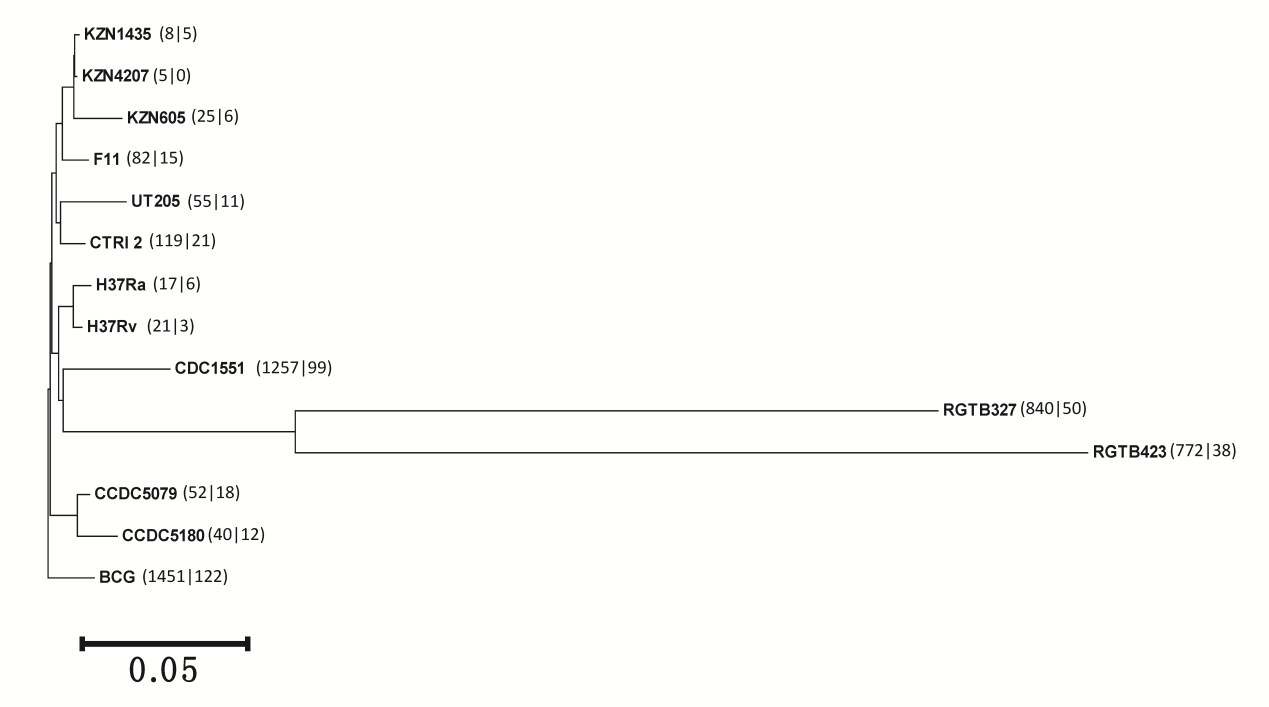


## Figure S5. Fifty-seven housekeeping genes are used in the phylogenetic analysis. The number next to the scale bars is the bootstrap confidence value. The scale bars represent the relative distances between different strains.

(Figure S5.pdf)

## Figure S6 - The distribution of original annotation overlaps in thirteen *M. tuberculosis* strains. The horizontal axis represents the gene orders ranked by overlap ratio, and the vertical axis represents overlap ratio. The largest overlap ratio in the original annotations is near 100%, and the threshold of overlap ratio in CCDC5180 is less than 20%.


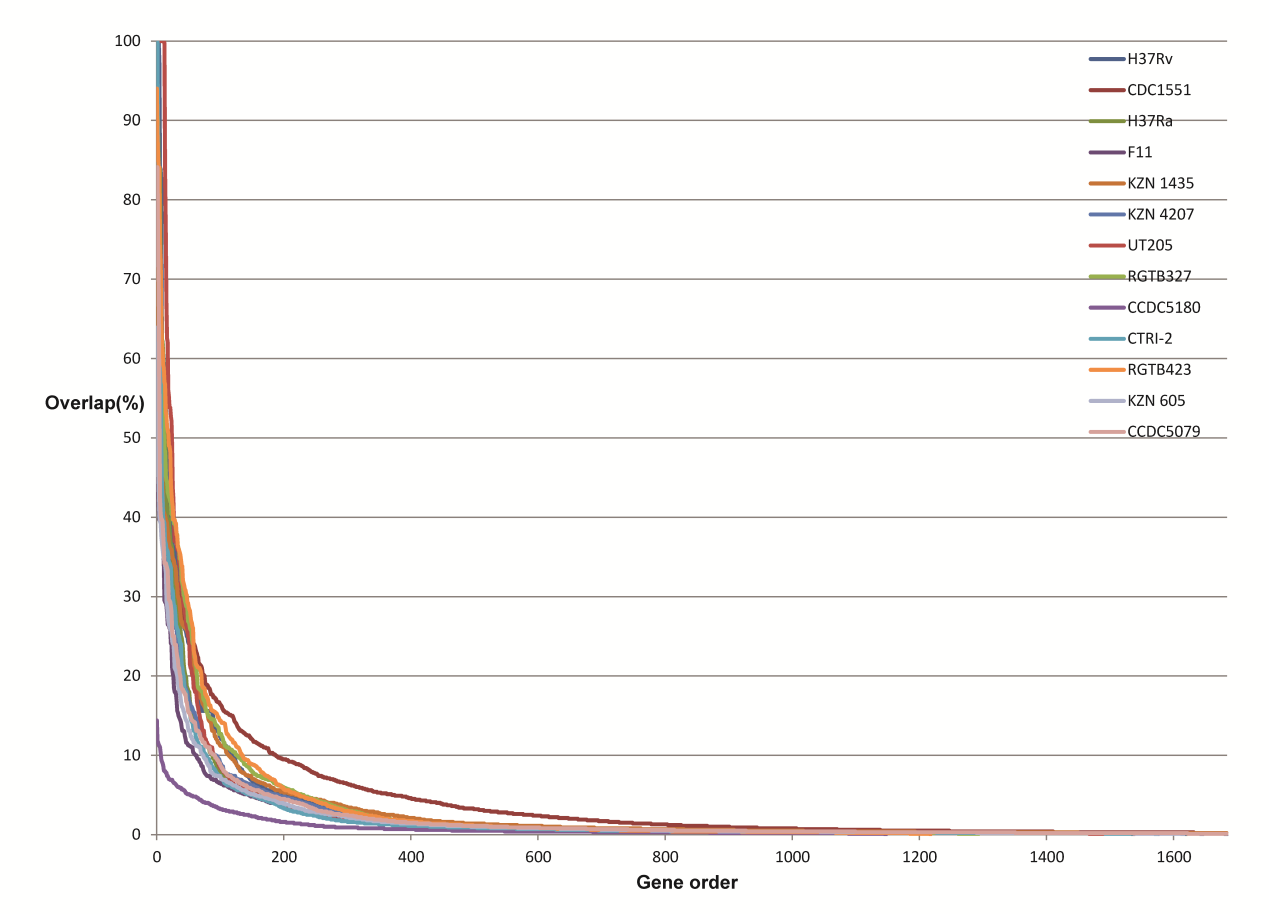


## Figure S7 - The distribution of overlaps after annotation refinement in thirteen *M. tuberculosis.* The horizontal axis shows the thirteen *M. tuberculosis* strain names, and the vertical axis shows the total gene count in each strain. The overlapped genes increase in every strain and both the number of overlapped genes and overlap ratio share similar distributions among the thirteen strains.


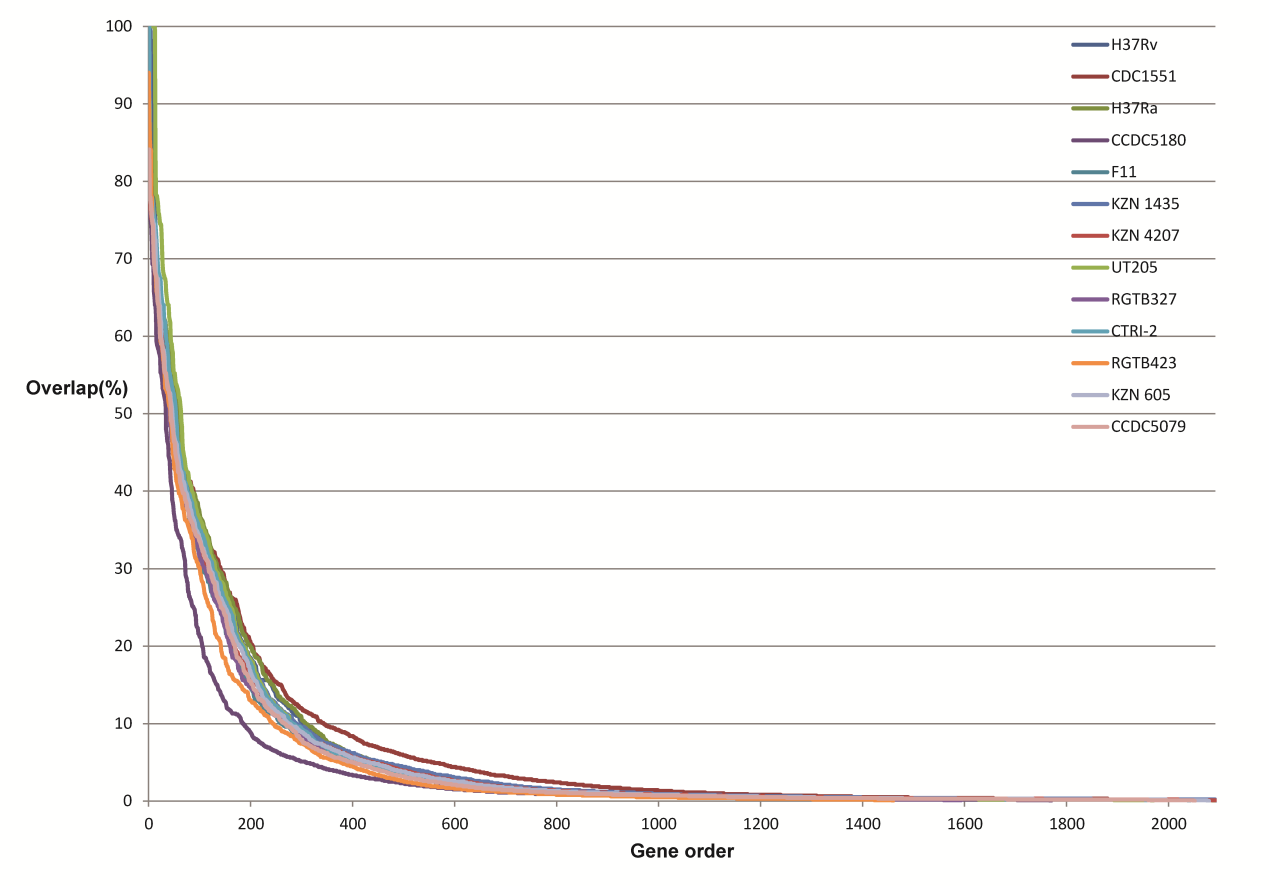

Supplement: Additional file 1: Table S1. — Enrichment analysis of unfixed COG groups from 13 M. tuberculosis strains. Figure S1. Examples of different degree (DD) calculations in two reduced GTNs. Figure S2. Distribution of genes from COG4118 (black bar) in H37Ra and H37Rv. Figure S3. The counts of unfixed COG groups at different levels of DD in thirteen M. tuberculosis. Figure S4. A rooted phylogenetic tree constructed with orthologs assigned by the COGs of 13 M. tuberculosis stains and the outgroup M. bovis BCG. Figure S6. The distribution of original annotation overlaps in thirteen M. tuberculosis strains. Figure S7. The distribution of overlaps after annotation refinement in thirteen M. tuberculosis. [file 12864_2015_1259_MOESM1_ESM.docx]
