# Supplementary material for: Comparing Mycobacterium tuberculosis genomes using genome topology networks
Source: BMC Genomics. 2015 Feb 14;16(1):85. doi: 10.1186/s12864-015-1259-0 (PMC4342819; doi:10.1186/s12864-015-1259-0)

Additional file 2: Figure S5. Fifty-seven housekeeping genes are used in the phylogenetic analysis. The number next to the scale bars is the bootstrap confidence value. The scale bars represent the relative distances between different strains.

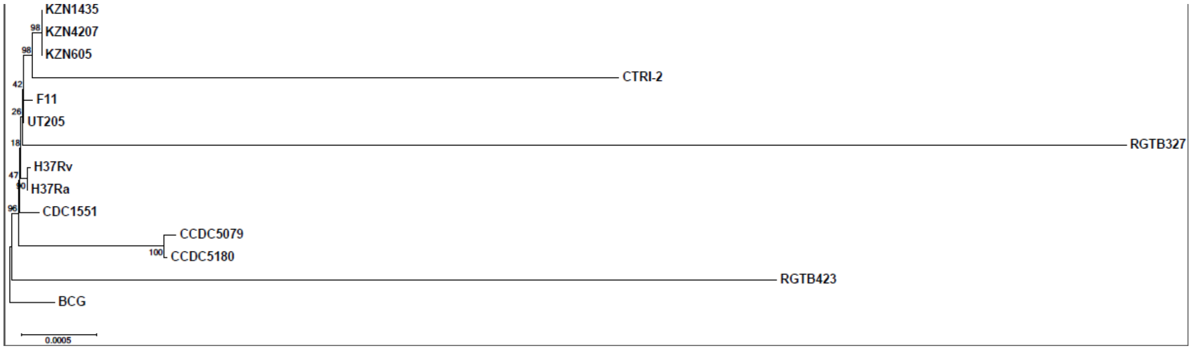

Supplement: Additional file 2: Figure S5. — Phylogenetic analysis of fifty-seven housekeeping genes shared within 13 M. tuberculosis stains and M. bovis BCG. [file 12864_2015_1259_MOESM2_ESM.pdf]
